# Primary Tonsillar Small Cell Neuroendocrine Carcinoma with Bone Metastases 

**DOI:** 10.22038/IJORL.2022.58137.3001

**Published:** 2022-07

**Authors:** Nik-Mohd-Syahrul-Hafizzi Awang, Ali Haron, Baharudin Abdullah

**Affiliations:** 1 *Department of Otorhinolaryngology-Head and Neck Surgery, School of Medical Sciences, Universiti Sains Malaysia, 16150 Kubang Kerian, Kelantan, Malaysia.*; 2 *Department of Otorhinolaryngology-Head and Neck Surgery, Hospital Raja Perempuan Zainab II, 15200 Kota Bharu, Kelantan, Malaysia.*

**Keywords:** Bronchopulmonary, Neuroendocrine carcinoma, Parapharyngeal space, Tonsillar hypertrophy, Thoracolumbar vertebrae

## Abstract

**Introduction:**

Small cell neuroendocrine carcinoma (NEC) that arises from the tonsil is a particularly rare head and neck carcinoma. This kind of neoplasm mainly originated from the bronchopulmonary area; however, there were reported cases of extrapulmonary areas. The prognosis is poor as the tumour is an aggressive tumour and have a high risk of metastasis.

**Case Report:**

We experienced a patient presented with painless right neck swelling and hard tonsillar hypertrophy for past six month. Computer tomography showed the tumour extended to the parapharyngeal space and metastasized to the thoracolumbar vertebras. The intraoral biopsy of the tonsil confirmed primary small cell neuroendocrine carcinoma of the tonsil. The clinical presentation, radiological imaging, histopathological investigations, and methods of treatment are discussed.

**Conclusions:**

Due to the rarity of this disease, there is no definitive treatment yet for this disease. The physicians must thoroughly understand the nature and characteristic of the disease to find the best treatment. The latest discoveries in chemotherapy drugs and radiotherapy may improve the treatment modalities in the future.

## Introduction

A small cell neuroendocrine carcinoma (NEC) of the tonsil is a scarce form of head and neck cancer (1). The common tumours in the tonsillar area are squamous cell carcinoma, minor salivary gland tumours, lymphoma, melanoma, and sarcomas (2). Small cell NEC is most often found in the pulmonary area, accounting for 14% of all lung cancers. However, NEC also can arise from gastrointestinal, genitourinary and other regions of the body (3). 

The incidence of extrapulmonary account for 0.1% to 0.4%. Small cell NEC is a highly aggressive cancer. It carries a poor prognosis as it has a high mortality rate, and the treatment for this cancer is not yet fully established. Here, we described a scarce case of primary small cell NEC originated from the tonsil, its clinical features, histopathological and radiological findings and its treatment. 

## Case Report

A 52-year-old man was referred to the otorhinolaryngology department of the Hospital Raja Perempuan Zainab II for insidious and painless right neck swelling persisting for the previous six months. Physical examination revealed a non-tender right neck swelling measuring 8 cm x 5 cm and with a hard inconsistency at the lateral neck levels II and III. Intraoral examination showed bilateral tonsil grade III with a normal mucosa appearance. However, on palpation, the consistency of the right tonsil was found to be hard relative to normal tonsillar consistency. Nasoendoscopy revealed no abnormalities. 

**Fig 1:**
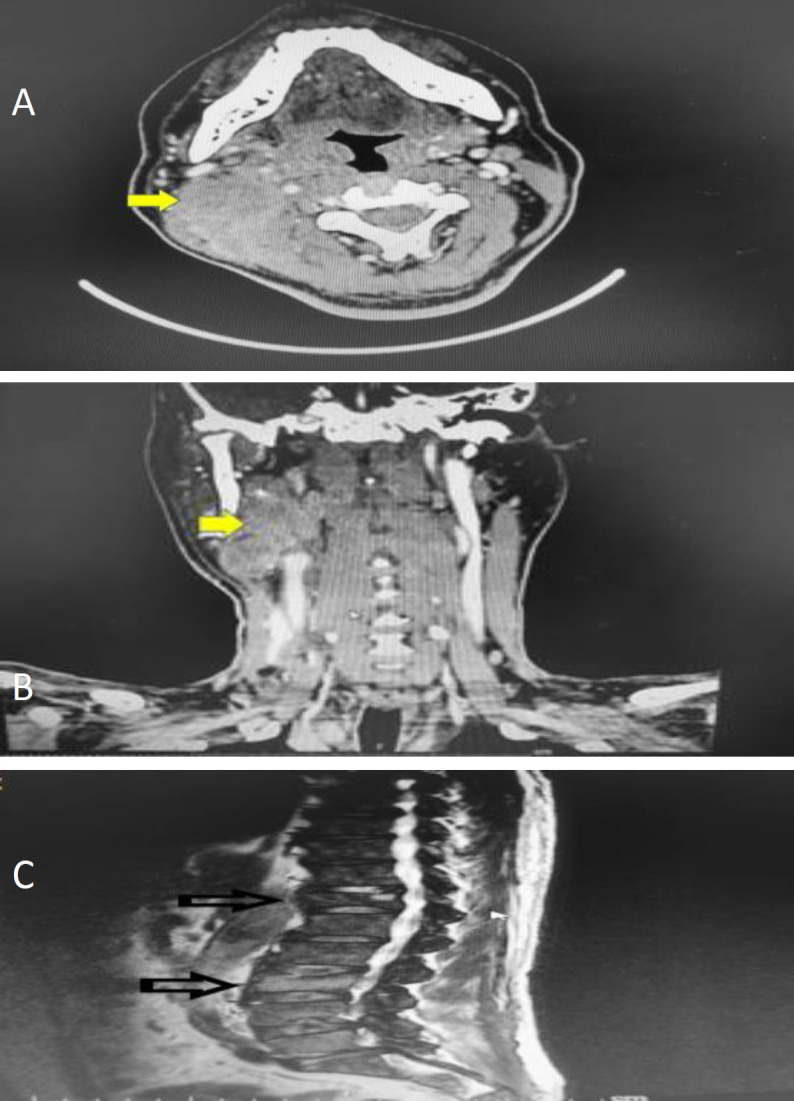
**A**. CT Neck (axial view) showed lesion at right tonsil extend to parapharyngeal region (yellow arrow), **B**. CT Neck (coronal view) showed lesion at right parapharyngeal region (yellow arrow), **C. **T2 sagittal view of the MRI of the thoracolumbar vertebrae showed pathological compression fracture at the level of T12 and L3 (black arrow)

A computer tomography (CT) scan of the neck found a lobulated, heterogeneously enhancing lesion with evidence of necrosis in the right cervical region measuring 3 cm x 6 cm x 7 cm and with an enlarged bilateral palatine tonsil on the right side. The plane of demarcation between the right lateral oropharyngeal wall and the parapharyngeal fat was obliterated (Figure 1). There was extensive bone metastasis with epidural involvement but no evidence of lung mass. Intraoral biopsy of the right tonsil and fine-needle aspiration of the right neck mass were performed in a clinical outpatient setting. Both investigations identified features of small cell neuroendocrine carcinoma, with the cells showing positive staining towards cytokeratin AE1/ AE3, CK7 and CD56. Full blood count indicated bicytopenia, and full blood pictures determined that it was due to metastatic bone marrow infiltration. Because of findings of distant bone metastasis in the CT scan, thoracolumbar magnetic resonance imaging (MRI) was performed. The MRI showed a pathological fracture at the levels of T12 and L3. Due to the advanced stage of the disease, no active surgical intervention was performed. The patient was referred to the oncology team for chemotherapy. The oncology team prescribed cisplatin-based chemotherapy (CCDP) for a total of 5 cycles and 35 fractions of radiotherapy to the neck area with a cumulative dose of 70 Grey. Upon completing the third cycle of chemotherapy and 15 fractions of radiotherapy, the patient showed significant improvement in his condition. However, the patient developed neutropenic sepsis with lung infection during subsequent treatments and succumbed to the disease.

## Discussion

Globally, the number of reported cases of primary small cell NEC in the head and neck is low. Three cases were initially reported in 1972 (1). The larynx is the most common NEC site in the head and neck, followed by the salivary glands, nasal cavity, and paranasal sinuses (3). The disease strikes most often between the fifth and seventh decades of life and is twice as prevalent in males than in females (4).

The main symptom of the NEC is a painless neck mass with or without throat pain and odynophagia. Physical examination may demonstrate asymmetrical tonsillar swelling or ulcerating tonsillar mucosa. However, some patients may present with a regular size and appearance of tonsillar mucosa. Thus, abnormal findings can be missed without proper palpation of the tonsil or radiological evaluation. Diagnostic tonsillectomy plays a significant role in the diagnostic workup of suspected malignancy of the head and neck (5). However, in this case, a punch biopsy of the tonsil was performed because the patient had thrombocytopenia and was at a high risk for bleeding, making tonsillectomy inappropriate. 

CT and MRI can be used to determine the size and infiltration of the tumour as well as to confirm that the tumour is not metastasis of another distant primary, especially from the lung. Positron emission tomography (PET) is the gold standard for determining the origin and epicentre of small cell neuroendocrine carcinoma in the head and neck (5). However, a PET scan was not done in this case due to limitations of the facility. Diagnosis of this tumour can be made based on clinical features, histopathological findings and radiological imaging. In a histopathological study, tumours observed under light microscopy have a small size, round-to-oval form, scant cytoplasm, finely granular nuclear chromatin and are devoid of nucleoli (6). 

Necrosis is common and widespread. Mitotic rates are high, averaging 80 mitoses per 2 mm^2^ area (7). In immunohistochemistry, the most useful markers are neural cell adhesion molecules (CD56), chromogranin and synaptophysin.CD56 will stain the tumour in approximately 90–100% of cases. However, CD56 staining is less specific for small cell carcinoma interpretation; thus, diagnosis needs to be correlated with morphological findings (8). In this case, the tumour cells were round-to-oval shaped with a high nuclear-to-cytoplasmic ratio. 

**Fig 2:**
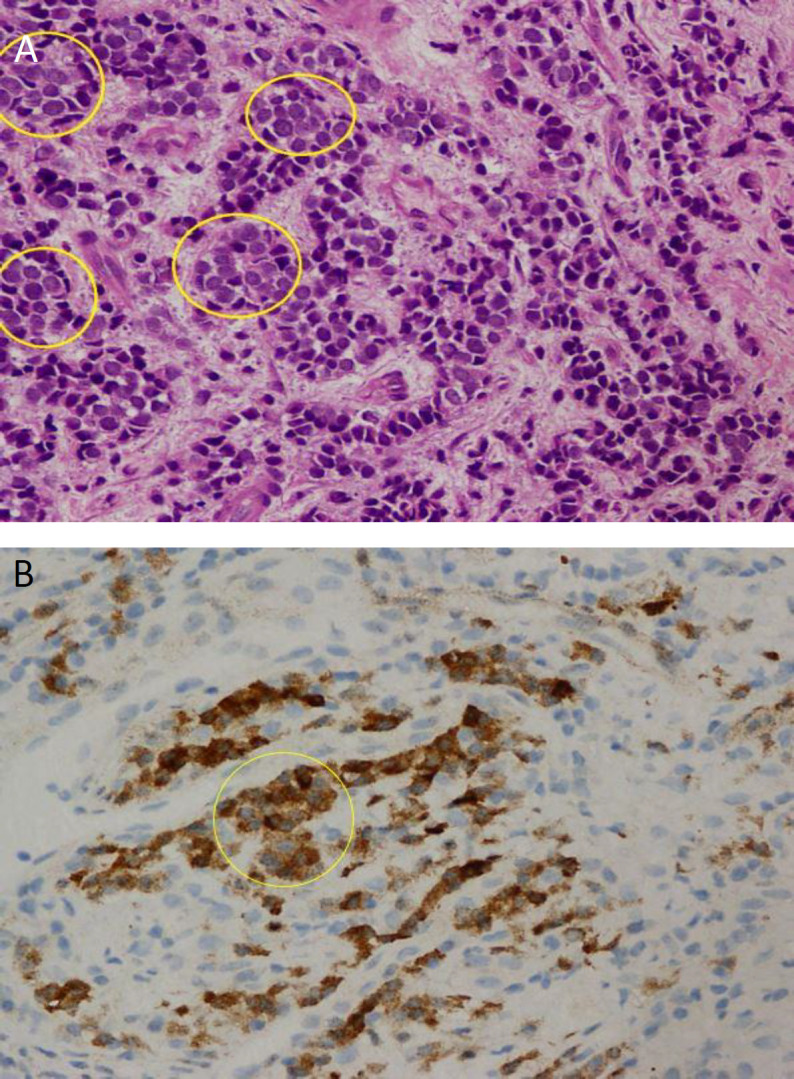
**A. **In light microscopy**, **the histological image showed the tumour cells with round to oval shape with high nuclear to cytoplasmic ratio (yellow circle).** B. **Immunohistochemical study shows that tumour cells positive towards CD56. (yellow circle)

The nuclei were finely dispersed with a salt-and-pepper chromatin pattern and the absence of nucleoli. Nuclear moulding was easily identified (Figure 2A). The tumour cells showed positive staining towards CKAE1AE3, CK7 and CD56 (Figure 2B).

Treatment for small cell NEC in the head and neck is not well established due to its rarity. However, various treatment modalities based on treatment for small cell NEC of the larynx and lung have been suggested, including radiotherapy, chemotherapy, surgical resection and combined modality approaches. A previous study reported that extrapulmonary small cell carcinoma responded well to both chemotherapy and chemoradiotherapy, similar to small cell carcinoma of the lung. However, a poor prognosis remains due to its strong metastatic potential (9). Thus, patients with small cell NEC should be considered for comprehensive chemotherapy to avoid early metastasis. The chemotherapy agents that have been approved to treat NEC are the topoisomerase I inhibitor irinotecan hydrochloride (CPT-11) and the alkylating agent cisplatin (CDDP). 

A recent study has proved that CPT-11 and CDDP may be used together to be an effective treatment for extrapulmonary small cell carcinoma of the oropharynx (9), while a few studies have shown that radiotherapy to the primary tumour site and neck produces a better outcome than surgery (10). Despite multimodality therapy, the outcomes for patients with NEC remain poor, as 66.7% experience recurrence or distant metastases, often dying from the disease within 2.5 years, with a median survival period of 18 months (10). 

Besides the local regional spreading to cervical lymph nodes, metastasizes of the tumour mostly to the liver, lungs, bones, brain, and skin. The concurrent radiotherapy and chemotherapy that our patient received managed to control the disease's progression. 

However, the immunocompromised state and the advanced disease spreading had led to a secondary infection that worsens his condition. Prognosis may be better if the patient was diagnosed earlier and at the early stage of the disease.

## Conclusions

In conclusion, small cell NEC of the tonsil is a rare head and neck carcinoma, which poses a significant challenge for the physicians. The prognosis is poor as the carcinoma is highly aggressive and can spread further to distant organs. The latest discoveries in chemotherapy drugs and radiotherapy may improve the treatment modalities in the future.
